# Vermin Riddance

**Published:** 1858-02

**Authors:** 


### VERMIN RIDDANCE.

The first step towards ridding a house of bugs, flies, rats, and reptiles is, to keep it scrupulously clean, from cellar to garret. But there are multitudes of housekeepers who are too lazy, ignorant, or thoughtless, to adopt this method; besides, some of our readers may be under the unfortunate necessity of moving into a house recently occupied by human pigs. Our humanity is stimulated to impart to them the following items of information; and they are particularly valuable, as they will obviate the necessity of keeping poisonous articles about the house, to the destruction of men as well as mice.

*Bedbugs* are effectually destroyed by washing infected places with a decoction of the common *Smart-weed* or "Water Pepper," called by Botanists *Pologonum punctatum.* Pour a pint of boiling water on a pint of the weed, cover it up, and let it cool. The liquor may be put on with a brush. The plant itself may be stuffed in cracks or corners.

